# Glutathione synthesis is essential for pollen germination in vitro

**DOI:** 10.1186/1471-2229-11-54

**Published:** 2011-03-26

**Authors:** Bernd Zechmann, Barbara E Koffler, Scott D Russell

**Affiliations:** 1 University of Graz, Institute of Plant Sciences, Schubertstrasse 51, 8010 Graz, Austria; 2 Graz University of Technology, Institute for Electron Microscopy and Fine Structure Research, Steyrergasse 17, 8010 Graz, Austria; 3 University of Oklahoma, Department of Botany and Microbiology, Samuel Roberts Noble Electron Microscopy Laboratory, 770 Van Vleet Oval, Norman, Oklahoma, 73019, USA

**Keywords:** Arabidopsis, auxin, gametophyte, glutathione, indole-3-acetic acid, pollen

## Abstract

**Background:**

The antioxidant glutathione fulfills many important roles during plant development, growth and defense in the sporophyte, however the role of this important molecule in the gametophyte generation is largely unclear. Bioinformatic data indicate that critical control enzymes are negligibly transcribed in pollen and sperm cells. Therefore, we decided to investigate the role of glutathione synthesis for pollen germination in vitro in *Arabidopsis thaliana *accession Col-0 and in the glutathione deficient mutant *pad2-1 *and link it with glutathione status on the subcellular level.

**Results:**

The depletion of glutathione by buthionine sulfoximine (BSO), an inhibitor of glutathione synthesis, reduced pollen germination rates to 2-5% compared to 71% germination in wildtype controls. The application of reduced glutathione (GSH), together with BSO, restored pollen germination and glutathione contents to control values, demonstrating that inhibition of glutathione synthesis is responsible for the decrease of pollen germination in vitro. The addition of indole-3-acetic acid (IAA) to media containing BSO restored pollen germination to control values, which demonstrated that glutathione depletion in pollen grains triggered disturbances in auxin metabolism which led to inhibition of pollen germination.

**Conclusions:**

This study demonstrates that glutathione synthesis is essential for pollen germination in vitro and that glutathione depletion and auxin metabolism are linked in pollen germination and early elongation of the pollen tube, as IAA addition rescues glutathione deficient pollen.

## Background

Glutathione is an important antioxidant and redox buffer in eukaryotes and most prokaryotes that fulfills many roles in plant metabolism and plant defense during abiotic and biotic stress conditions in the sporophyte [1], but its role remains largely unknown for the gametophyte. In the sporophyte, glutathione is involved in the detoxification of reactive oxygen species (ROS), redox signaling, the modulation of gene expression and in the regulation of enzymatic activities [extensively reviewed by 1]. Glutathione is also involved in the detoxification of xenobiotics, herbicides [2,3] heavy metals such as cadmium [4-8], and protects proteins from oxidation by a process called glutathionylation [9-11]. The importance of glutathione for plant growth and development is highlighted by the observation that impaired glutathione synthesis correlates with growth defects [12,13], and that the complete absence of glutathione synthesis results in a lethal phenotype [14]. Additionally, the redox state of glutathione is also important for plant growth and development. In non-stressed plants it occurs mainly in its reduced form (GSH), whereas during oxidative stress high amounts of oxidized glutathione (GSSG) can be formed. The occurrence of high amounts of GSSG correlates with reduced growth, dormancy, or cell death [15-17].

Glutathione synthesis takes place in two ATP-dependent steps triggered by enzymes that are encoded by single genes in Arabidopsis [18]. In the first step cysteine is linked with glutamate to form γ-glutamylcysteine. This reaction is triggered by γ-glutamylcysteine synthetase (GSH1). In the second step, glycine is linked to γ-glutamylcysteine by glutathione synthetase (GSH2) to form the final product glutathione. In Arabidopsis, these two steps seem to take place exclusively in plastids and the cytosol, which are therefore considered as the main centers of glutathione synthesis [18]. In the sporophyte, transcripts of GSH1 and GSH2 and the final product, glutathione, can be found in all major plant parts and in all cell organelles at different concentrations (Additional files 1 and 2) [19,20]. According to immunohistochemistry and quantitative transmission electron microscopy, the highest levels of glutathione have been detected in mitochondria and the lowest in plastids. Vacuoles contained glutathione only under certain conditions (e.g. high sulfur soil contents, high amounts of oxidized glutathione) [21,22], whereas glutathione could not be detected in cell walls [20]. Although the role and subcellular distribution of glutathione in the sporophyte are well defined, the necessity and role of glutathione in the gametophyte remain largely unexamined. Bioinformatic data cast doubt on the importance of glutathione metabolism in the male gametophyte, as both GSH1 and GSH2 are transcribed at negligible levels in pollen and sperm cells (Additional files 1 and 2) [23,24]. Nevertheless, due to the apparent sensitivity of microspores to mitochondrial damage induced by chronic oxidative stress [25], we decided to investigate the necessity and localization of glutathione in the gametophyte in order to reveal strategies for pollen to cope with ROS during tube germination and elongation.

In the current study, we investigate whether glutathione is essential for pollen germination, and if so, whether such pools of glutathione depend on new synthesis or an existing glutathione pool available in the pollen grains before the start of germination. The subcellular distribution of glutathione will also reveal if glutathione is distributed equally or shows compartment-specific adaptations as found in leaf and root cells [20].

In order to achieve this goal we studied pollen germination rates and the subcellular distribution of glutathione in pollen grains of wildtype plants treated with and without buthionine sulfoximine (BSO). BSO is known to inhibit the first enzyme of the glutathione synthesis pathway thus leading to a strong decrease in glutathione levels [26-31]. Additionally, we studied pollen germination rates and the subcellular distribution of glutathione in the pollen grains of the *pad2-1 *mutant, which has a mutation of the first enzyme of the glutathione synthetic pathway thus reducing the content of glutathione by 80% compared to the wildtype [19,32]. Differences in glutathione contents between the wildtype and the *pad2-1 *mutant should give detailed insight into the roles of temporary and permanent glutathione depletion on pollen germination in vitro.

## Results

### Pollen germination

**Figure 1:**
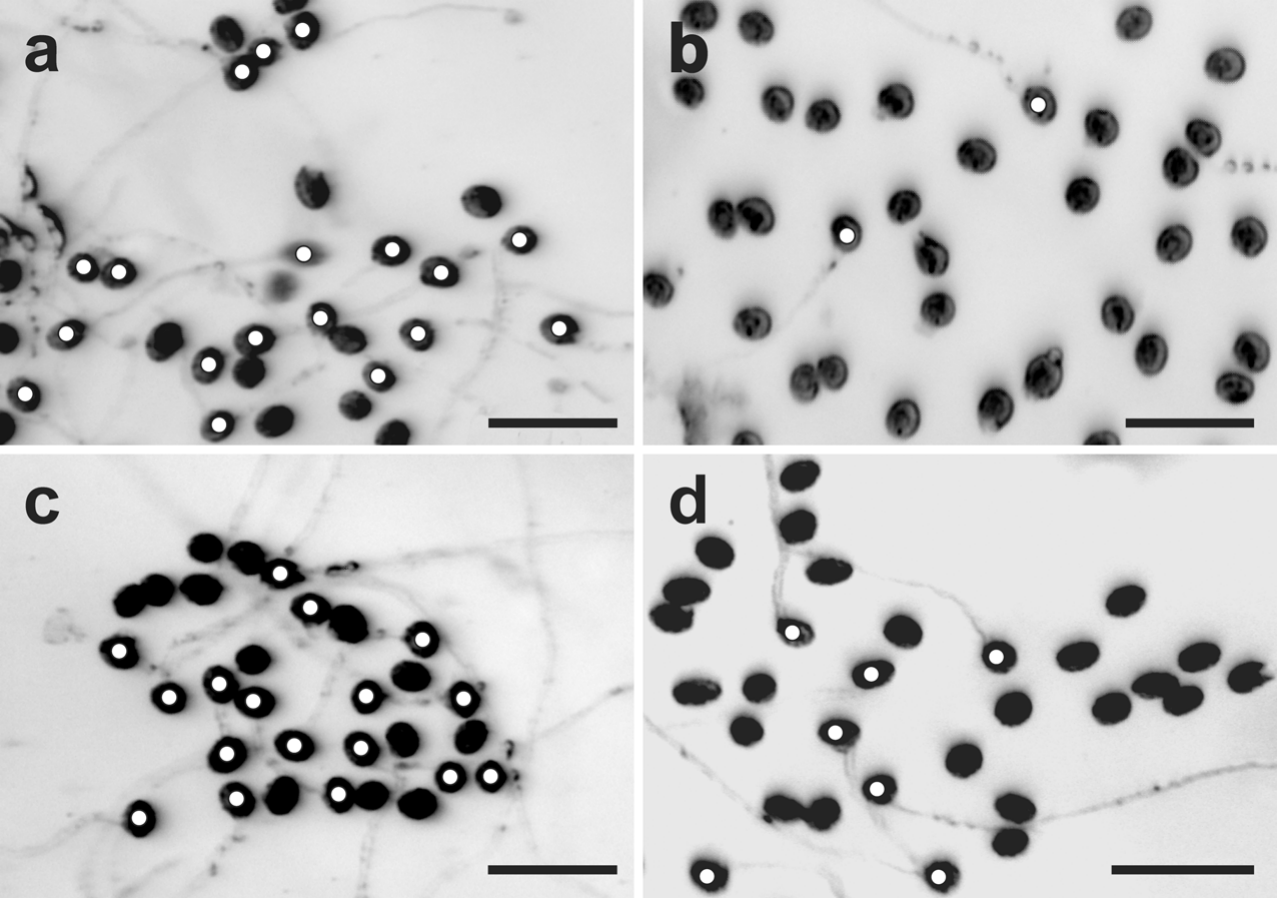
**Images showing the effect of BSO and GSH treatment on pollen germination in Col-0**. Light microscopical images show *Arabidopsis thaliana *accession Col-0 pollen grains after 16 h incubation on solidified pollen germination media containing (a) no GSH and BSO (control), (b) 0.1 mM BSO, (c) 0.1 mM BSO and 1 mM GSH, and (d) 0.1 mM BSO and 3 mM GSH. Original images were inverted and germinating pollen grains were marked with white circles for better visualization. Bars = 100 μm.

**Figure 2:**
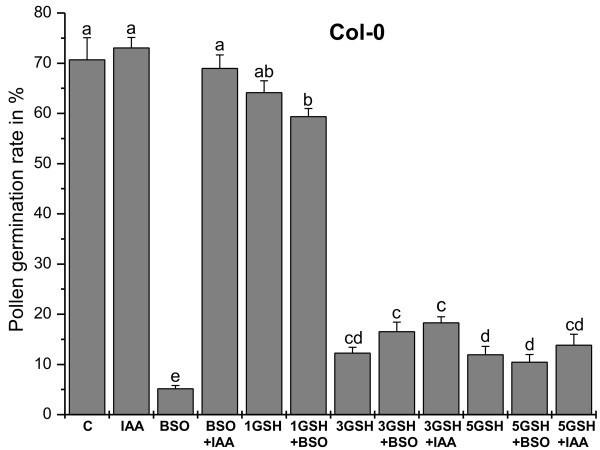
**Statistical analysis of the effects of BSO, GSH, and IAA treatment on pollen germination rate in Col-0**. Graph shows *Arabidopsis thaliana *accession Col-0 pollen germination rates (%) after 16 h incubation on solidified pollen germination media containing different concentrations of BSO (0.1 mM), GSH (1, 3, or 5 mM) and IAA (22.8 μM) for 16 hours. Data represent means and standard errors. Different lowercase letters indicate significant differences (P < 0.05) analyzed with the Kruskal-Wallis test followed by post-hoc comparison according to Conover. N > 2000 pollen grains per treatment from 3 or more independent experiments.

**Figure 3:**
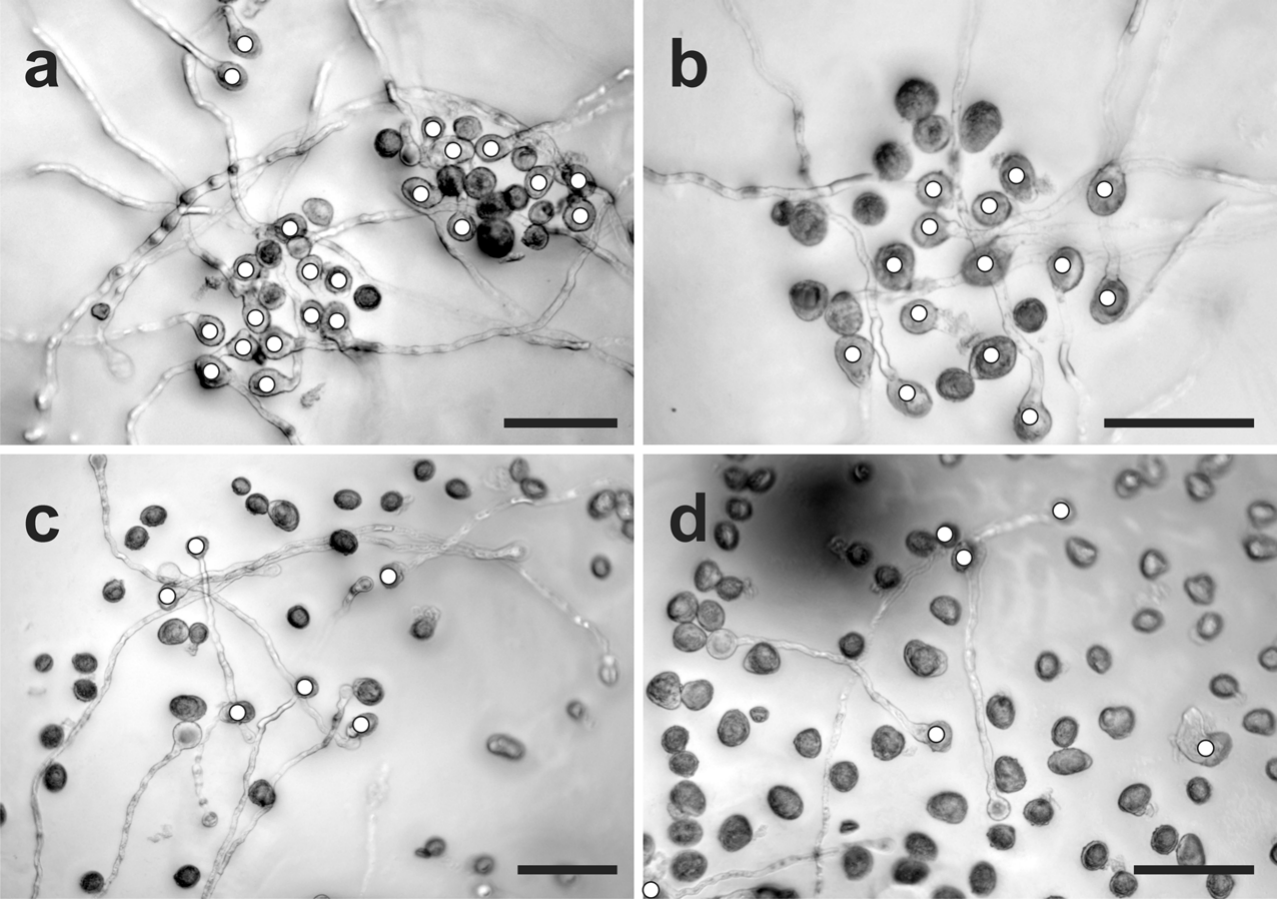
**Images showing the effect of IAA treatment on pollen germination**. Light microscopical images show pollen grains of *Arabidopsis thaliana *accession Col-0 (a, b) and the *pad2-1 *mutant (c, d) after 16 h incubation on solidified pollen germination media containing a, c) 22.8 μM IAA and b, d) 0.1 mM BSO and 22.8 μM IAA. Pollen grains were marked with white circles for better visualization. Bars = 100 μm.

Wildtype pollen grains showed a germination rate of 71% when grown on germination medium without GSH and BSO (Figure 1 and Figure 2). Treatment of 0.1 mM BSO, which depletes glutathione, decreased pollen germination rate to about 5%. To examine whether glutathione depletion may be linked with decreased levels of auxin, which is one of the most important plant hormones for pollen tube growth [33,34], we have applied 22.8 μM indole-3-acetic acid (IAA) together with BSO in the growth media. The addition of IAA to growth media containing BSO restored pollen germination rate to levels similar to the control (69%; Figure 2 and Figure 3). Similar levels were reached (73%) when pollen germination was performed on pollen germination media containing only IAA (Figure 2 and Figure 3). The addition of 1 mM GSH to the media containing 0.1 mM BSO restored pollen germination rate to 60%. A similar rate (64%) was found when wildtype pollen grains (without BSO) were allowed to germinate on media containing 1 mM GSH. Pollen germination rate was reduced to 12% when higher concentrations of GSH (3 mM and 5 mM) were added to the growth medium (Figure 2). A similar germination rate (16% and 11%, respectively) was observed after pollen grains were transferred onto pollen germination media containing 0.1 mM BSO and either 3 mM or 5 mM GSH (Figure 1 and Figure 2). The addition of IAA to the pollen media containing 3 mM and 5 mM GSH did not significantly change pollen germination (Figure 2). Higher BSO concentrations (1.5 mM and 5 mM) had similar inhibitory effects on pollen germination rate as observed using media containing 0.1 mM BSO (Additional file 3). Nevertheless, the addition of 1 mM GSH only partially restored pollen germination rate (29% and 12%) when added to 1.5 mM and 5 mM BSO, respectively. The addition of 3 mM GSH to pollen germination media containing 1.5 mM and 5 mM led to pollen germination at rates of 14% and 5%, respectively (Additional file 3).

**Figure 4:**
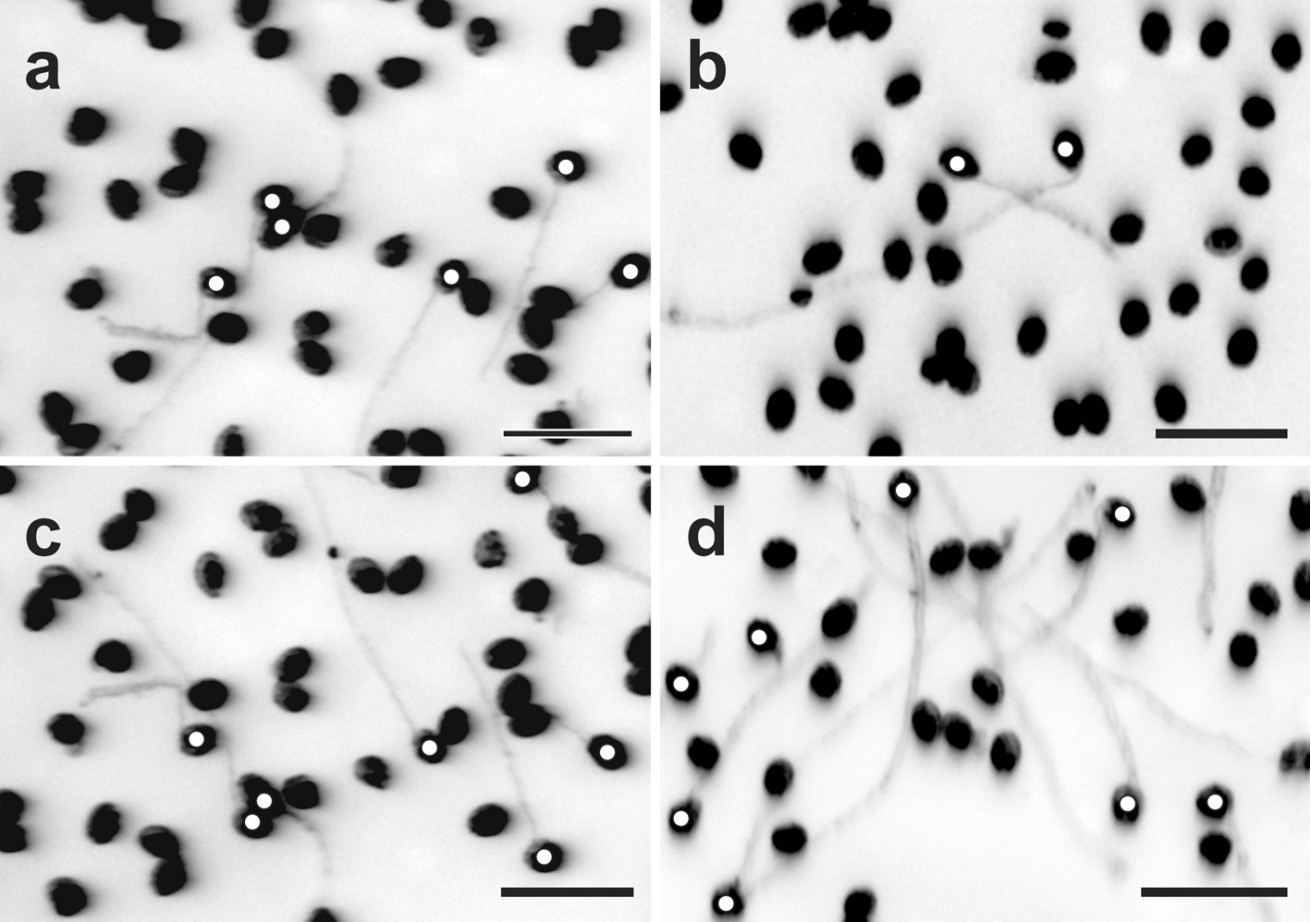
**Images showing the effects of BSO and GSH treatment on pollen germination in *pad2-1***. Light microscopical images show pollen grains of the *Arabidopsis thaliana *mutant *pad2-1 *after 16 h incubation on solidified pollen germination media containing (a) no GSH and BSO (control), (b) 0.1 mM BSO, (c) 0.1 mM BSO and 1 mM GSH, and (d) 0.1 mM BSO and 3 mM GSH. Original images were inverted and germinating pollen grains were marked with white circles for better visualization. Bars = 100 μm.

**Figure 5:**
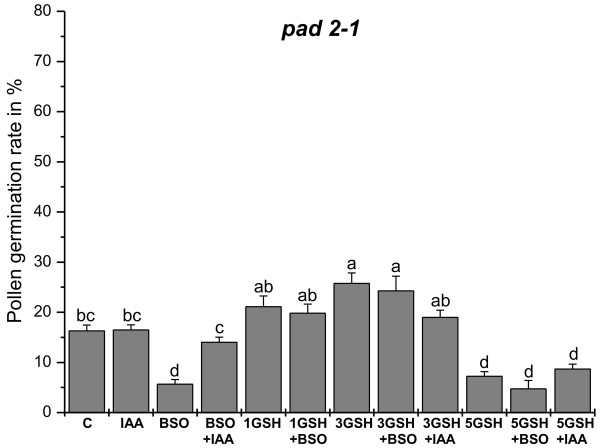
**Statistical analysis of the effects of BSO, GSH, and IAA treatment on pollen germination rate in *pad2-1***. Graph shows pollen germination rates (%) of pollen obtained from the *Arabidopsis thaliana *mutant *pad2-1 *after 16 h incubation on solidified pollen germination media containing different concentrations of BSO (0.1 mM), GSH (1, 3, or 5 mM) and IAA (22.8 μM) for 16 hours. Data represents means and standard errors. Different lowercase letters indicate significant differences (P < 0.05) analyzed with the Kruskal-Wallis test followed by post-hoc comparison according to Conover. N > 2000 pollen grains per treatment from 3 or more independent experiments.

Pollen germination of the *pad2-1 *mutant was around 16% on medium without GSH and BSO (Figure 4 and Figure 5). Similar levels were reached (16%) when pollen germination was performed on pollen germination media containing IAA (Figure 3 and Figure 5). Treatment of 0.1 mM BSO decreased pollen germination rate to 6%. The addition of auxin to growth media containing BSO restored pollen germination rates to levels similar to the control (14%; Figure 3 and Figure 5). Adding 1 mM and 3 mM GSH to the medium containing 0.1 mM BSO increased pollen germination rate to 16% and 25%, respectively (Figure 4 and Figure 5). Similar levels were found when 1 mM GSH and 3 mM GSH were added to pollen germination media without BSO (21% and 26%, respectively). The addition of 5 mM GSH to medium containing 0.1 mM BSO decreased pollen germination rates to 5%, which was similar to the germination rate of pollen grown on medium with the addition of 5 mM GSH (Figure 5). The addition of IAA to the growth medium containing 3 mM and 5 mM GSH did not affect pollen germination and was similar to the germination rate of pollen grown on 3 mM and 5 mM GSH alone (Figure 5). The addition of higher BSO concentrations (1.5 mM and 5 mM) decreased pollen germination rates to 6% and 4%, respectively (Additional file 4). The addition of 1 mM and 3 mM GSH to media containing 1.5 mM BSO restored pollen germination rates to 23% and 10%, respectively. Adding 1 mM GSH and 3 mM GSH to media containing 5 mM BSO increased the pollen germination rates of the *pad2-1 *mutant to 11% and 6%, respectively (Additional file 4).

### Glutathione labeling

**Figure 6:**
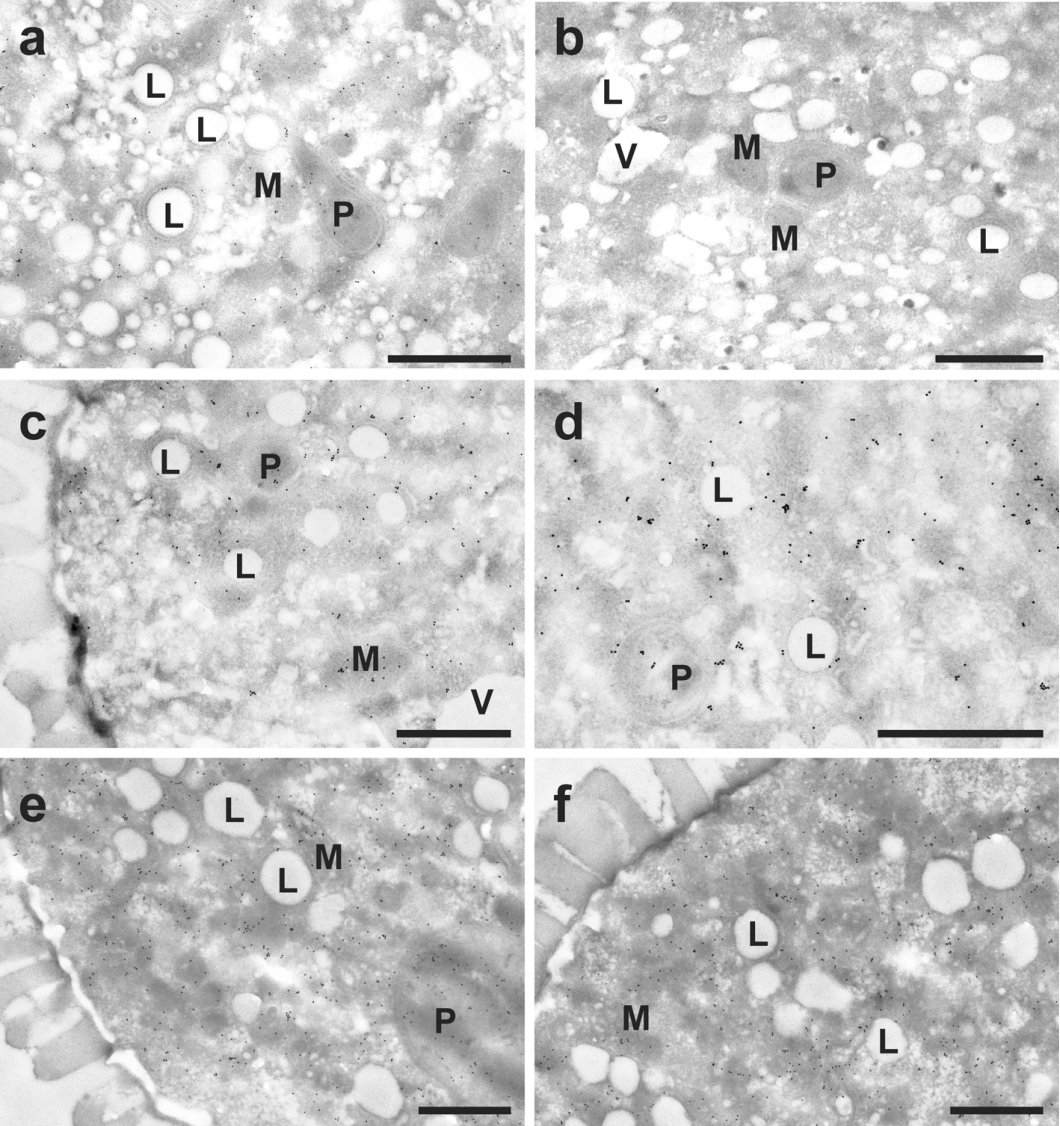
**Transmission electron micrographs showing the subcellular distribution of glutathione in Col-0 pollen grains**. Gold particles bound to glutathione could be found evenly distributed in plastids (P), mitochondria (M), and the cytosol but not in lipid bodies (L), vacuoles (V) and cell walls of pollen grains obtained from *Arabidopsis thaliana *accession Col-0. Pollen grains were grown on solidified pollen germination medium for 5 hours with either (a) no GSH and BSO (control), (b) 0.1 mM BSO, (c) 1 mM GSH, (d) 0.1 mM BSO and 1 mM GSH, (e) 3 mM GSH, and (f) 3 mM GSH and 0.1 mM BSO prior to fixation. Bars = 1 μm.

**Figure 7:**
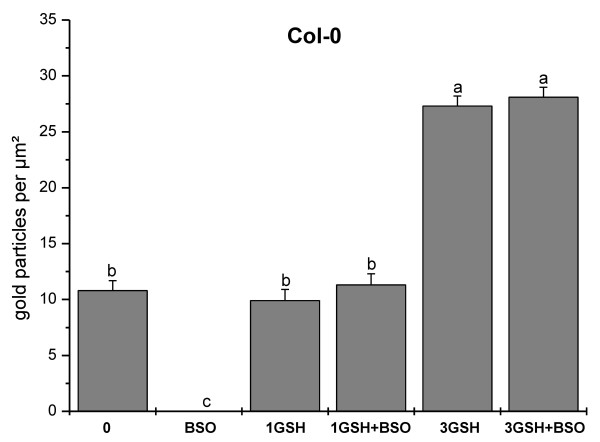
**Statistical analysis of the subcellular distribution of glutathione in pollen grains of Col-0**. Total amount of gold particles bound to glutathione per μm^2 ^in pollen grains of *Arabidopsis thaliana *accession Col-0. Pollen grains were incubated for 5 hours on solidified pollen germination media containing different concentrations of BSO (0.1 mM) and GSH (1 or 3 mM). Data represent means and standard errors. Different lowercase letters indicate significant differences (P < 0.05) analyzed with the Kruskal-Wallis test followed by post-hoc comparison according to Conover. N > 20 pollen grains per treatment from 2 independent experiments.

**Figure 8:**
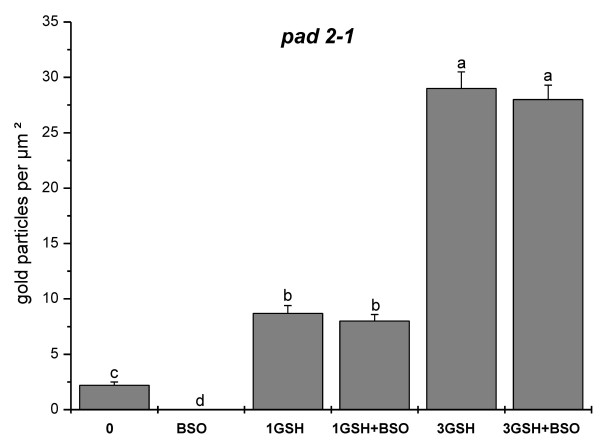
**Statistical analysis of gold particle density in pollen grains of pad2-1**. Total amount of gold particles bound to glutathione per μm^2 ^in pollen grains of the *Arabidopsis thaliana *mutant *pad2-1*. Pollen grains were incubated for 5 hours on solidified pollen germination media containing different concentrations of BSO (0.1 mM) and GSH (1 or 3 mM). Data represent means and standard errors. Different lowercase letters indicate significant differences (P < 0.05) analyzed with the Kruskal-Wallis test followed by post-hoc comparison according to Conover. N > 20 pollen grains per treatment from 2 independent experiments.

**Figure 9:**
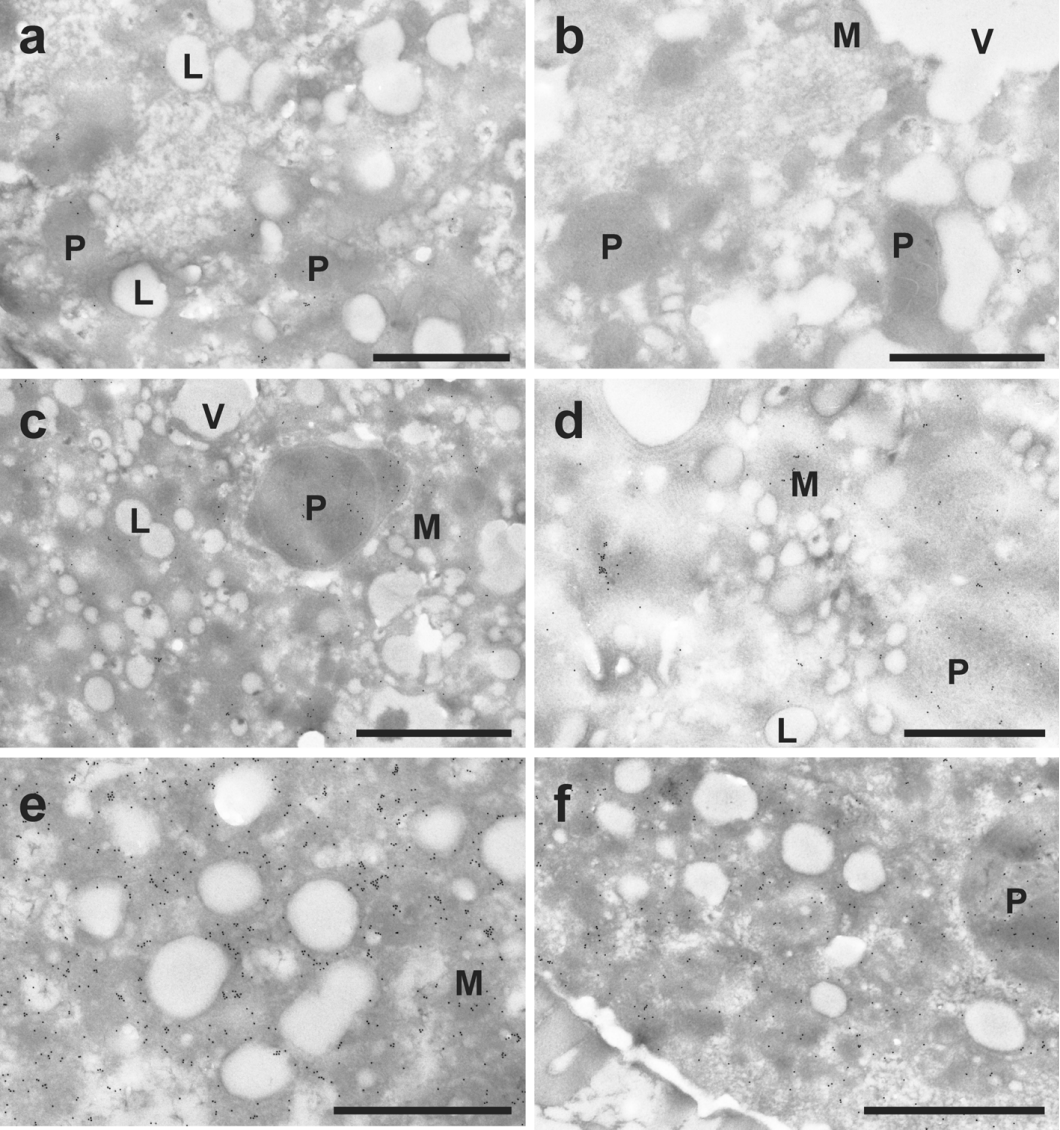
**Transmission electron micrographs showing the subcellular distribution of glutathione in pollen grains of pad2-1**. Gold particles bound to glutathione could be found evenly distributed in plastids (P), mitochondria (M), and the cytosol but not in lipid bodies (L), vacuoles (V) and cell walls in pollen obtained from the *Arabidopsis thaliana *mutant *pad2-1*. Pollen grains were grown on solidified pollen germination medium for 5 hours with either (a) no GSH and BSO (control), (b) 0.1 mM BSO, (c) 1 mM GSH, (d) 0.1 mM BSO and 1 mM GSH, (e) 3 mM GSH, and (f) 3 mM GSH and 0.1 mM BSO prior to fixation. Bars = 1 μm.

Immunogold particles localized to glutathione were found in all cell compartments except cell walls and vacuoles (Figure 6). Gold particle density was much higher in pollen obtained from wildtype plants than in *pad2-1 *mutants (10.8 and 2.2 gold particles per μm^2^, respectively; Figure 7 and Figure 8). Mitochondria, plastids, nuclei and the cytosol contained equally dense quantities of gold particles bound to glutathione (Figure 6 and Figure 9). Gold particle density was similar in wildtype pollen grains which were allowed to germinate on medium without GSH and BSO (10.8 gold particles per μm^2^) and on media containing 1 mM GSH with and without 0.1 mM BSO (11.3 and 9.9 gold particles per μm^2^, respectively). An increase in glutathione contents in wildtype pollen between 161% and 153% was observed when 3 mM GSH was added to the media with or without 0.1 mM BSO, respectively (Figure 7). The treatment of pollen grains from Col-0 and the *pad2-1 *mutant with BSO decreased gold particle density to background levels (<0.1 gold particles per μm^2^; Figures 6, 7, 8, 9).

Pollen grains from the *pad2-1 *mutant contained lower glutathione-labeling density (2.2 gold particles per μm^2^) than wildtype (Figure 8), whereas the addition of 1 mM GSH and 3 mM GSH to the germination media increased glutathione contents to values similar to pollen grains from wildtype plants (8.7 and 29 gold particles per μm^2^, respectively). Similar gold particle densities were reached when 0.1 mM BSO was added to GSH (Figure 8).

## Discussion

The results of this study clearly demonstrate that glutathione synthesis is essential for pollen germination in vitro. Despite negligible levels of GSH1 and GSH2 transcripts occurring in pollen and sperm cells [23,24], glutathione was found to be clearly present and active in the male gametophyte. Pollen germination rates of wildtype pollen, used as controls in the current study (71%), were similar to those observed in previous studies under similar conditions [35]. Inhibition of glutathione synthesis by BSO decreased the pollen germination rate to about 5%, which could be correlated with the absence of glutathione specific labeling in our immunogold assays. BSO inhibits the first enzyme (GSH1) of the glutathione synthesis pathway, which leads to a decrease or complete absence of glutathione in leaves [26-31]. In pollen grains of the *pad2-1 *mutant, in which glutathione synthesis is distorted by a single point mutation in GSH1, decreasing glutathione content to ~20% of wildtype [32] a similar result is observed. Pollen germination of the *pad2-1 *mutant was reduced to about 16%, and correlated with an 80% reduction in glutathione specific immunolabeling when compared to the wildtype. A further decrease of pollen germination rate to 5% was accomplished by the addition of BSO to the growth media. Similar results have also been observed during germ tube development in *Candida albicans*. Similarly, strong depletions in GSH contents induced by 1-chloro-2,4 dinitrobenzene were correlated with very significant reductions in germ tube formation capacity and severe cell mortality [36]. The addition of 1 mM GSH to growth media containing BSO, could restore pollen germination rate and glutathione specific labeling to values similar to the controls in pollen from both wildtype plants and the *pad2-1 *mutant. Thus, these results clearly demonstrate that glutathione synthesis and the availability are essential for pollen germination and demonstrate the importance of glutathione not only for plant development but also for pollen germination and tube growth. Additionally, these results showed that pollen grains where glutathione synthesis was inhibited by BSO were able to import glutathione from the growth medium by unknown mechanisms. This could be important considering that glutathione uptake transporters have not been identified yet at the plasma membrane and that negligible transcripts levels of GSH1 and GSH2 were found in pollen and sperm cells [23,24].

The *pad2-1 *GSH1 point mutant showed a much lower pollen germination rate (71% vs. 16%, respectively), which presumably correlates with a much lower glutathione content (-80%) when compared to the wildtype. As the *pad2-1 *mutant accumulates only about 20% glutathione levels of the wildtype [32], these results demonstrate that glutathione contents in pollen grains strongly depend on adequate glutathione availability in the plant. Compartment-specific differences (e.g., accumulation of glutathione in mitochondria), as observed in leaves and roots of the *pad2-1 *mutants [19], could not be detected in pollen grains where glutathione concentrations were found to be distributed in all typically labelled cell compartments equally. A slightly higher germination rate of *pad2-1 *pollen (about 25%) could be accomplished by the addition of 3 mM GSH to the growth media, which still displayed far below the normal germination rate of wildtype control pollen grains (71%) and was similar to the germination rate achieved when pollen grains from the wildtype were treated with the same GSH concentration (16%). The addition of 5 mM GSH decreased pollen germination rate in both wildtype and *pad2-1 *mutants to 12% and 6%, respectively, demonstrating that high levels of GSH negatively affect the ability of pollen grains to germinate. It has been demonstrated recently that the treatment of roots with high levels of GSH can cause severe ultrastructural alterations [31,37]. Additionally, it has been demonstrated that in GSH-overexpressing tobacco plants, elevated glutathione biosynthetic capacity in the chloroplasts paradoxically increased oxidative stress, leading to severe ultrastructural alterations within chloroplasts and to their ultimate degeneration, eventuating in the death of the cell [38]. Even though such ultrastructural changes have not been observed during the present study, treatment of pollen grains with high levels of GSH (e.g. 3 and 5 mM GSH) could potentially inhibit pollen germination (e.g., by changing the internal redox status). The application of BSO together with IAA, one of the most important hormones regulating pollen tube growth [33,34] restored the rate of pollen germination in the *pad2-1 *mutant to about 14%, which was similar to levels achieved by untreated pollen of the *pad2-1 *mutant used as control. The same experiment restored the rate of pollen germination in the wildtype of BSO-treated pollen to over 60%. Thus, these results demonstrate that low glutathione levels in pollen grains of the *pad2-1 *mutant must have altered their ability to germinate in the long term. It has been demonstrated recently that the accumulation of ROS in mitochondria was found to be critical for proper pollen development, as sterile pollen grains showed decreased pools of ATP and NADH and lower activity of mitochondria DNA [25]. As glutathione is essential for the detoxification of ROS/H_2_O_2 _in plants it seems likely that the low germination rates of pollen grains from the *pad2-1 *mutants were caused by low glutathione levels in the plant and pollen grains during pollen development in the stamen. Nevertheless, pollen grains are thought to possess resistance against or ability to downregulate production of stigma-associated ROS/H_2_O_2 _(e.g. by antioxidants) in order to germinate on and penetrate through the stigma [39]. As pollen germination of *pad2-1 *pollen grains could be only partly increased by the addition of 1 and 3 mM GSH (from 14% to 16% and 25%, respectively) but never reached rates of the wildtype (71%) these results also suggest that insufficient glutathione is present without activity of GSH1 to permit normal rates of germination. Thus, we can conclude that sufficient glutathione contents are required during pollen development and also during pollen germination for proper pollen germination of pollen from the *pad2-1 *mutant.

Since a reduction of root growth induced by the depletion of GSH is caused by the inhibition of auxin transport [40,41], we tested if inhibited pollen germination by BSO can be restored by treatment with IAA, which is one of the most important plant hormones for pollen tube growth [33,34]. Results of this study demonstrated that the application of BSO together with IAA diminished the deleterious effects of BSO and led to pollen germination rates similar to that of control pollen. The addition of IAA alone or together with GSH did not have such affects. Thus, we can conclude that glutathione depletion in pollen grains triggered disturbances in auxin metabolism which are linked with inhibition of pollen germination induced by BSO treatment.

## Conclusions

Summing up, it can be concluded that glutathione synthesis is essential for pollen germination in vitro. Additionally, it was demonstrated that low glutathione levels in the *pad2-1 *mutant decreased their ability to germinate caused by disturbances most probably during pollen development. The mechanisms behind the reduction of pollen germination induced by glutathione depletion could be correlated with disturbances in auxin metabolism which still have to be explored in more detail.

## Methods

### Plant material

After stratification for 4 days at 4°C, seeds of *Arabidopsis thaliana *[L.] Heynh. Ecotype Columbia (Col-0) originally obtained from the European Arabidopsis stock centre (NASC; Loughborough, UK), and the glutathione deficient mutant line *pad 2-1 *were grown on soil in greenhouse conditions with approximately 16/8 h day/night photoperiod. Day and night temperatures were 22°C and 18°C, respectively. The relative humidity was 60% and the plants were kept at 100% relative soil water content. Light intensity varied between 120-150 μmol m^-2 ^s^-1^.

### Determination of pollen germination

Four to six weeks after flowering, pollen was harvested from about 20 different plants for each experiment and transferred onto pollen germination medium mounted on glass slides, which were kept and prepared in moist chambers at 22°C. Pollen germination medium was always prepared fresh with double distilled water and contained 5 mM CaCl_2_, 1 mM MgSO_4_, 5 mM KCl, 0.01 mM H_3_BO_3_, 10% sucrose and 1.5% agarose. The pH-value was adjusted to 7.5 with a 1 M NaOH solution. In addition, different concentrations of reduced glutathione (GSH), buthionine sulfoximine (BSO), and indole-3-acetic acid (IAA) were added to the germination media. Pollen germination media contained either (a) no GSH and BSO (control), (b) 22.8 μM IAA, (c) 0.1 mM BSO, (d) 0.1 mM BSO and 22.8 μM IAA, (e) 1 mM GSH, (f) 1 mM GSH and 0.1 mM BSO, (g) 3 mM GSH, (h) 3 mM GSH and 0.1 mM BSO, (i) 3 mM GSH and 22.8 μM IAA, (j) 5 mM GSH, (k) 5 mM GSH and 0.1 mM BSO, and (l) 5 mM GSH and 22.8 μM IAA. In preliminary studies additional concentrations of BSO and GSH were tested (1.5 mM BSO and 5 mM BSO with or without the addition of 1 mM and 3 mM GSH) in order to evaluate the ideal BSO and GSH concentrations for the proposed experiments. IAA concentration was chosen according to previous studies which demonstrated that the addition of 22.8 μM (4 mg L^-1^) IAA stimulated pollen tube growth most effectively [33,34]. Therefore, 100 mM stock solutions of GSH, BSO and IAA respectively, were prepared and small aliquots of these solutions were added to the pollen germination media to reach the final concentration of GSH, BSO, and IAA. The pH-value of the media was adjusted to 7.5 with a 1 mM NaOH-solution. After transferring pollen on the germination media, grains were allowed to germinate in the dark at 22°C in a temperature controlled incubator. Slides were either examined for pollen germination rates under a Zeiss Stemi SV11 or an Olympus Provis AX 70 microscope (Olympus, Life and Material Science Europa GmbH, Hamburg, Germany) 16 hours later. Digital images were taken of several randomly chosen areas on the slides containing pollen grains and the amount of pollen that germinated was determined with the help of the image analysis software Olympus Cell D (Olympus, Life and Material Science Europa GmbH, Hamburg, Germany).

### Sample preparation for electron microscopy

Pollen grains were allowed to germinate on solidified pollen germination media containing different concentrations of GSH and BSO for 5 hours. Then they were covered with 2.5% low melting agarose and transferred in the fixative solution after the agarose was solidified (within 30 seconds). For electron microscopical analysis pollen grains were fixed in 2.5% paraformaldehyde and 0.5% glutaraldehyde in 0.06 mM phosphate buffer (pH 7.5) containing 10% sucrose for 45 minutes. Samples were washed in buffer 4 times 15 minutes and dehydrated in increasing concentrations of acetone (50%, 70%, and 90%) for 2 times 10 minutes for each step. Infiltration was carried out with increasing concentrations of LR-White resin (30%, 50%, and 70%) mixed with 90% acetone with a minimum of 3 hours per step. Samples were then infiltrated with 100% LR-White resin for 4 hours and embedded in fresh resin for 48 hours at 50°C. Ultrathin sections (80 nm) were cut with a Reichert Ultracut S ultramicrotome (Leica, Microsystems, Vienna, Austria).

### Cytohistochemical investigations

Immunogold labeling of glutathione was conducted using ultrathin sections mounted on coated nickel grids and labeled with the Leica EM IGL automated immunogold labeling system (Leica, Microsystems, Vienna, Austria) according to Zechmann et al. and Zechmann and Müller [19,20]. For cytohistochemical analysis, samples were blocked with 2% bovine serum albumin (BSA) in phosphate buffered saline (PBS, pH 7.2) for 20 min at room temperature. The sections were then treated with the primary antibody against glutathione (anti-glutathione rabbit polyclonal immunoglobulinG [IgG]; Millipore Corp., Billerica, MA, U.S.A.) diluted 1:50 in PBS for 2 h. After short rinses in PBS (3 times 5 min) the samples were incubated with a 10 nm gold-conjugated secondary antibody (goat anti rabbit IgG; British BioCell International, CardiV, UK) diluted 1:50 in PBS for 90 min. After short washes in PBS (3 times 5 min) and distilled water (2 times 5 min) labeled grids were either immediately observed in a Philips CM10 transmission electron microscope or post stained with uranyl-acetate (2% dissolved in double distilled water) for 15 s. Post staining with uranyl acetate was applied to facilitate the distinction of different cell structures enabling a clearer identification of the investigated organelles.

### Quantitative analysis of immunogold labeling

Micrographs of randomly photographed immunogold labeled sections of pollen grains were digitized and gold particles were counted automatically using the software package Cell D using the particle analysis tool (Olympus, Life and Material Science Europa GmbH, Hamburg, Germany). A minimum of 20 sectioned pollen grains from two independent experiments were analyzed for gold particle density. The obtained data were recorded as the number of gold particles per μm^2^. For all statistical analyses the non-parametric Kruskal-Wallis test followed by a post-hoc comparison according to Conover was used. P < 0.05 was considered as significant.

## Abbreviations

ATP: adenosine triphosphate; BSO: buthionine sulfoximine; BSA: bovine serum albumin; IAA: indole-3-acetic acid; GSH: reduced glutathione; GSSG: oxidized glutathione; PBS: phosphate buffered saline; ROS: reactive oxygen species.

## Authors' contributions

BZ conceived of the study and participated in its design and coordination, carried out the electron and light microscopical work and drafted the manuscript. BK participated in electron and light microscopical studies, and performed quantitative and statistical analysis of the data. SDR participated in the design of the study and its coordination and helped to draft the manuscript. All authors read and approved the final manuscript.

## Supplementary Material

Additional file 1 **γ-glutamyl-cysteine synthetase (At4g23100), reported by available Affymetrix 24K Arabidopsis genomic microarray data at Genevestigator**. Click here for file

Additional file 2 **Glutathione synthetase (At5g27380), reported by available Affymetrix 24K Arabidopsis genomic microarray data at Genevestigator**. Click here for file

Additional file 3 **Effect of BSO (buthionine sulfoximine) and GSH (reduced glutathione) treatment on pollen germination rate**. Graph shows *Arabidopsis thaliana *accession Col-0 pollen germination rates (%) after 16 h incubation on solidified pollen germination media containing different concentrations of BSO (1.5 mM) and GSH (1 or 3 mM) for 16 hours. Data represent means and standard errors. Different lowercase letters indicate significant differences (P < 0.05) analyzed with the Kruskal-Wallis test followed by post-hoc comparison according to Conover. N > 2000 pollen grains per treatment from 3 or more independent experiments. Click here for file

Additional file 4 **Effect of BSO (buthionine sulfoximine) and GSH (reduced glutathione) treatment on pollen germination rate**. Graph shows pollen germination rates (%) of pollen obtained from the *Arabidopsis thaliana *mutant *pad2-1 *after 16 h incubation on solidified pollen germination media containing different concentrations of BSO (1.5 mM) and GSH (1 or 3 mM) for 16 hours. Data represent means and standard errors. Different lowercase letters indicate significant differences (P < 0.05) analyzed with the Kruskal-Wallis test followed by post-hoc comparison according to Conover. N > 2000 pollen grains per treatment from 3 or more independent experiments. Click here for file
