# Supplementary figures and images for: Glutathione synthesis is essential for pollen germination in vitro
Source: BMC Plant Biol. 2011 Mar 26;11:54. doi: 10.1186/1471-2229-11-54 (PMC3078877; doi:10.1186/1471-2229-11-54)

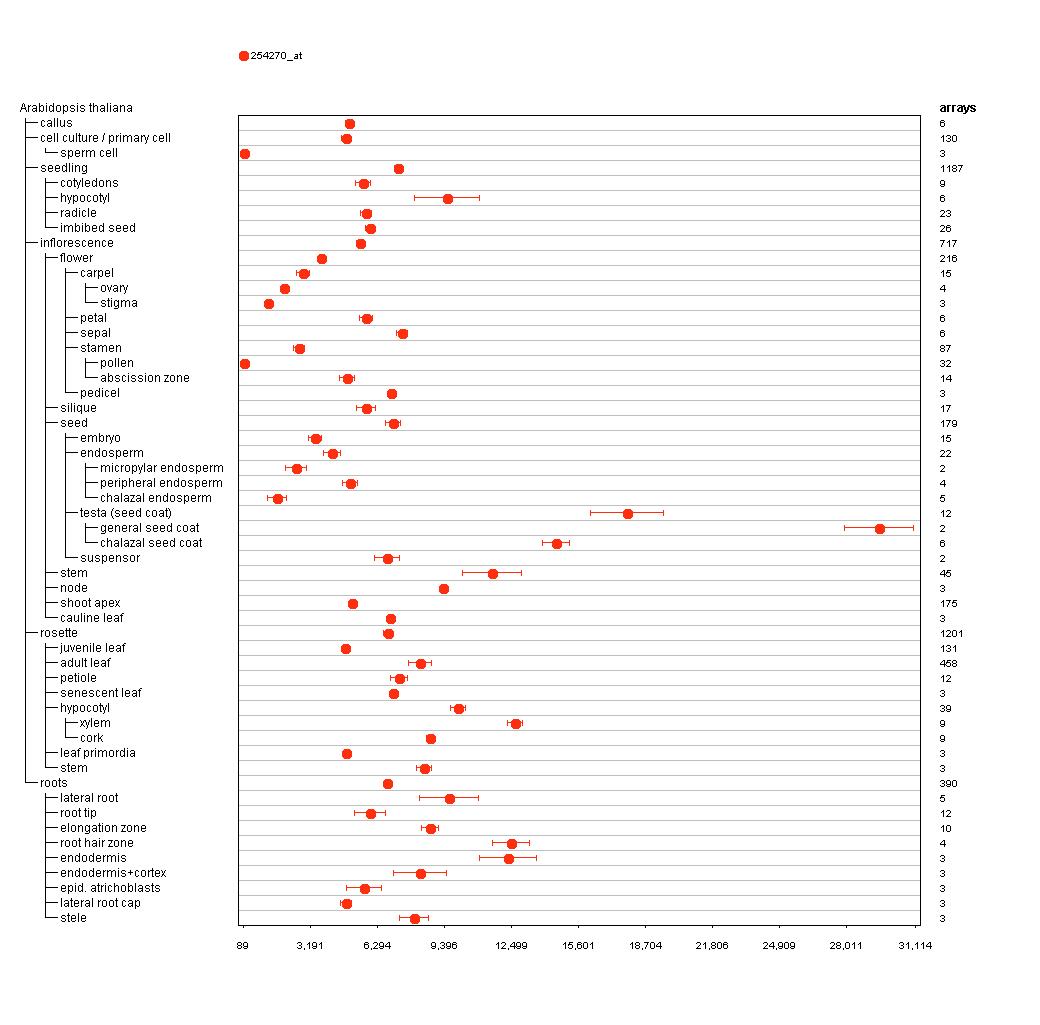

Supplement: Additional file 1 — γ-glutamyl-cysteine synthetase (At4g23100), reported by available Affymetrix 24K Arabidopsis genomic microarray data at Genevestigator. [file 1471-2229-11-54-S1.DOC]

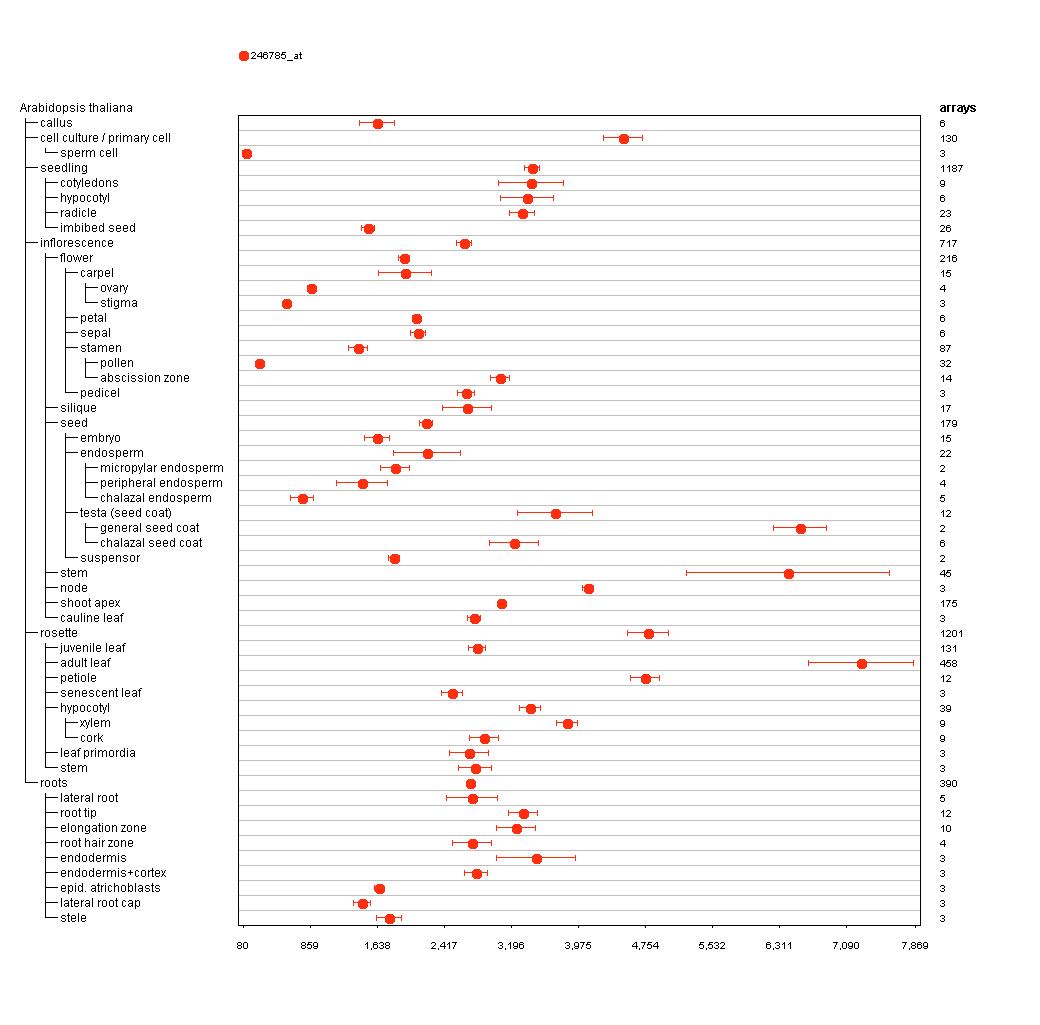

Supplement: Additional file 2 — Glutathione synthetase (At5g27380), reported by available Affymetrix 24K Arabidopsis genomic microarray data at Genevestigator. [file 1471-2229-11-54-S2.DOC]
