# Supplementary material for: Glutathione synthesis is essential for pollen germination in vitro
Source: BMC Plant Biol. 2011 Mar 26;11:54. doi: 10.1186/1471-2229-11-54 (PMC3078877; doi:10.1186/1471-2229-11-54)
Supplement: Additional file 4 — Effect of BSO (buthionine sulfoximine) and GSH (reduced glutathione) treatment on pollen germination rate. Graph shows pollen germination rates (%) of pollen obtained from the Arabidopsis thaliana mutant pad2-1 after 16 h incubation on solidified pollen germination media containing different concentrations of BSO (1.5 mM) and GSH (1 or 3 mM) for 16 hours. Data represent means and standard errors. Different lowercase letters indicate significant differences (P < 0.05) analyzed with the Kruskal-Wallis test followed by post-hoc comparison according to Conover. N > 2000 pollen grains per treatment from 3 or more independent experiments. [file 1471-2229-11-54-S4.DOC]

Additional File 4
