# Supplementary material for: Glutathione synthesis is essential for pollen germination in vitro
Source: BMC Plant Biol. 2011 Mar 26;11:54. doi: 10.1186/1471-2229-11-54 (PMC3078877; doi:10.1186/1471-2229-11-54)
Supplement: Supplementary file 3 [file 1471-2229-11-54-S3.DOC]

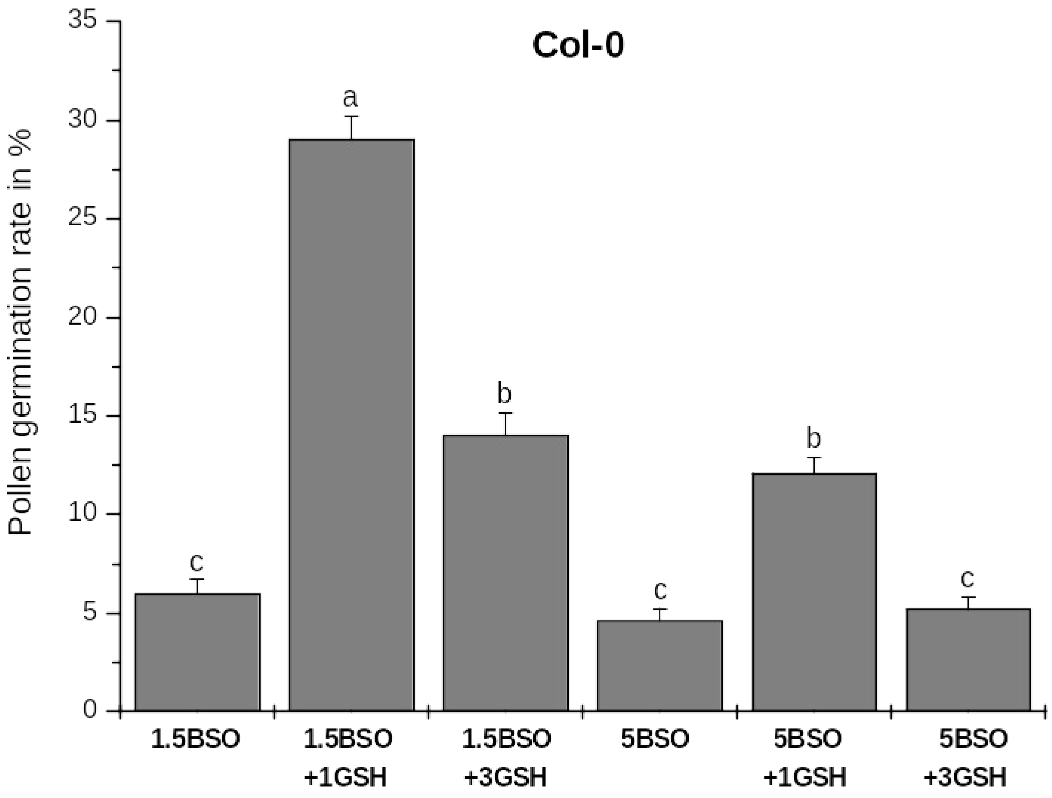
Col-0
Pollen germination rate in %
35
30
25
20
15
10
5
0
a
b
b
c
c
c
1.5BSO
1.5BSO +1GSH
1.5BSO +3GSH
5BSO
5BSO +1GSH
5BSO +3GSH
